# Sanitary Reform

**Published:** 1851-08

**Authors:** 


### SANITARY REFORM.

Physicians have not a deeper interest in sanitary science than other members of society, but they are expected to take the lead in its advancement and diffusion. They are expected to be acquainted with its principles and ready to instruct communities how it may be applied to the preservation of health and life. It is not enough that they cure disease when it comes; they are looked to for the means of preventing it. Life cannot be indefinitely extended, but it is undeniable that thousands die before their time, and tens of thousands languish on sick beds through causes that might be averted. It is to the medical profession that the eyes of men are turned for a cure of this evil.

Sanitary improvement stands forth conspicuously among the great reform movements of our age. It has made most progress in England, Germany, and France, but it is beginning to attract attention in our country. The reports of the Sanitary Commissions cannot fail to create a general interest in the matter—they show, indeed, an annual, unnecessary waste of human life that staggers belief, and must lead to reform. These reports contain not merely the year's births, marriages, and deaths, but give the date and cause of death, the occupation, age, sex, habitation, and locality of those who have died; in a word contain the sanitary history of vast communities and millions of individuals, for a series of years, by which we are enabled to compare the healthiness of various occupations and classes, and the mortality of different cities. The results which they bring out are very striking. For example, it appears from the English Registration Reports, that in about one-half of England, in towns as well as in the country, the rate of mortality is less than two per cent., while in many of its principal cities it is nearly three per cent. In London, the annual mortality is one in 39.10; in Sheffield, one in 29.28; in Liverpool, one in 34.92; in Leeds, one in 35.44; and in the whole of England, one in 45.8, or 2.18 per cent. Now, why this difference? This waste of life is dependent upon sanitary causes which are easily pointed out; and which human ingenuity and foresight might readily obviate. Filth, crowded, badly-ventilated houses, with their necessary accompaniments—these explain it nearly all.

An able writer in the North American Review, for July, furnishes some statistics on this head, which place the subject in the clearest light. He makes a comparison of three different sections of Boston, and nothing could more forcibly show the influence of locality upon human life. In the first district, comprising the best of Boston, the number of deaths for 1850 was one in 74.7 of the inhabitants, or 1.3 per cent. The next district is composed entirely of newly made land, with streets narrow, houses small, and drainage and sewerage imperfect. The inhabitants are principally mechanics and trades people, intelligent, and "as attentive to the laws of hygiene as the average of any community." Here, we learn, the mortality was one in 52.7 of the inhabitants, or 1.9 per cent.—not a high rate, certainly, but much higher than that of the first district. The third district is thus described:—

"It comprises Broad, Cove, and Sea streets. These streets are situated near the wharves. They are built principally upon made land, and have numerous blind alleys, or *cul-de-sacs*, leading from them. The streets and alleys are badly drained, and crowded with an overflowing population. A large number of the houses have no means of sewarage whatever, and all their refuse of every description stagnates about the yards, spreading on every side poisonous exhalations, laden with disease and death. A majority of the houses contain several families, and some of them have no less than nine or ten. Even the cellars of the houses are often inhabited. In some instances, one cellar leads to another, and this to a third, a sort of dungeon, all inhabited by human beings of both sexes and every age."

The mortality of this district was *one in* 17.6 *of the population*, or 5.65 per cent., and this, as the writer just quoted observes, "in a year remarkable for its healthiness." Of the population of this district 2,738 were foreigners, and only 75 Americans. This fact explains, in part, the appalling mortality, for the excesses of such a population and their neglect of personal cleanliness are prolific sources of disease; but after making all due deductions on this score much remains to be accounted for by the character of their dwellings; and it is just here that the public authorities should interpose. Ought any city to tolerate such a plague spot as that just described? Such wretched hovels ought to be condemned as public nuisances, and if not abated in any other way, purified by fire. It would be better for a city that a conflagration should sweep over such squares of filth and wretchedness than that they should remain to diffuse all around their pestilential atmosphere.

Boston exhibits a strong case, but there can be no doubt that all large cities furnish examples similar in kind. Let any one walk through the narrow alleys of any large town, and he will discover causes enough to explain the disproportionate mortality with which they are visited. But as there is a cause, so there is a remedy for all this; not simple, not easily applied, but complicated, difficult as the evil is vast. It requires for its application the joint wisdom, address, and energy of the medical profession and our municipal authorities. The public mind must be enlightened, and the science is to come from our profession. Medical men are demonstrating that the annual mortality of most communities is far greater than it need be; they are indicating the sources of this needless waste of human life. It is for those vested with the authority to apply the remedy. The indispensable requisites to health are an ample supply of good water, pure air, and light, with sufficient drainage and sewerage, and large exercise grounds. In a broad land like ours, why should any of these be denied to the poorest of the people?

This is a subject which medical men ought to take all proper occasions to press upon public attention. Y.

